# Book Review

**Published:** 1910-06

**Authors:** 


**Publications of the Research Defence Society.** Selected by the Committee. Pp. xv, 216. London: Macmillan and Co. 1909.—Even these twelve collected publications afford testimony sufficiently convincing to anyone with an open mind as to the claims of British Scientific Research, involving experiments on animals, to rank with the most humane of philanthropic efforts. The opening address of Lord Cromer, and the evidence of Lord Justice Fletcher Moulton before the Royal Commission are invaluable to the lay reader, because the value and justification of experimental research is plainly set forth by laymen who are able to apply the faculty of criticism developed to a high degree, while the contributions of others, such as those of Professor Osler on "Yellow Fever and Malaria," and Sir David Bruce on "The

Extinction of Malta Fever," will be appreciated and understood by both lay and medical readers.

**Index-Catalogue of the Library of the Surgeon-General's Office, United States Army.** Second Series. Vol. XIV. Q—Rzehak. Pp. 829. Washington: Government Printing Office. 1909.—The present volume of this most useful work contains 10,019 author titles, 4,065 subject titles of separate books and pamphlets, and 31,370 titles of articles in periodicals. It shows that even a new therapeutic agent like radium is already possessed of a considerable literature. The librarian is to be congratulated on the regularity with which these volumes appear, for the labour expended in the preparation of this indispensable work must be extremely great.

**Deuteronomy Smith.** By a Student of Medicine. Pp. 68. Edinburgh: E. and S. Livingstone.—This little book is the story of a medical student at Edinburgh, told in a style meant to imitate the stately language of the Old Testament. It is profusely illustrated by rather amusing sketches of the hero's progress and adventures. The tale has no special plot, and its only claim upon one's notice is the method of narration, which no doubt will cause merriment to some of those who have no objection to a paraphrase of a great classic—a form of entertainment which, personally, we do not much enjoy.

**Roller Skating for Health and Pleasure.** By J. WILLIAMS (professional and expert), and a Doctor of Medicine (amateur and enthusiast). Pp. 62. Bristol: John Wright & Sons Ltd. 1910.—This is a small manual written in praise of roller skating, and as a guide to beginners. The expert has made himself "responsible for the facts, whilst to the amateur has fallen the duty of transcribing these facts into words" (Preface). The illustrations are good, and indicate the kind of recreation to be obtained at a skating rink, no less than five of them being vivid representations of various tumbles; whilst others represent some of the phases of social intercourse that may be enjoyed. Some of the photographs are charming. A strong case is made out for the healthiness of this form of amusement, and one fully agrees with the medical author when he claims that it is better to skate than "to sit for most of the day with one's legs under a desk or in a drawing-room chair." This latter position must certainly be irksome. The effects on the muscles and joints is stated to be excellent, and we are reminded that "in the back there are 26 bones, 75 joints, 150 ligaments and about 290 muscles"; so there is really a chance for almost anyone to twist something, and surgeons need not take a gloomy view of the general outlook. As a guide to roller skating the book deserves praise for its concise and clear directions and for the useful diagrams. We could wish that our friend M.D. had made some suggestion as to an improvement in the atmosphere ot these rinks. We have, whilst watching and taking part in this exercise, found the air very oppressive, and this feeling was not, we believe, entirely due to the number of people, including M.D. himself, who fell upon us.

**The "Wellcome" Photographic Exposure Record and Diary.** 1910.—This new edition of this very useful little pocket-book is quite up to the standard of its predecessors, and contains several new features, such as "improved methods of obtaining wide variations in colour of sulphide-toned prints," and attention is drawn once again to the wide range of colour obtainable on lantern slides by varying the exposure and the strength of the developer. Both novice and expert will find this pocket-book full of valuable information, and we can cordially recommend it to the notice of every photographer.

**A System of Medicine.** Edited by Sir CLIFFORD ALLBUTT, K.C.B., M.D., and HUMPHRY DAVY ROLLESTON, M.D. Vol. IV, Part II. Pp. xii, 566. London: Macmillan and Co. Ltd. 1908.—It seems a little peculiar to have a separate volume in *A System of Medicine* devoted to subjects so surgical as diseases of the throat, nose and ear. In the original edition of the *System* the articles on affections of the nose and throat were in vol. iv, which contained also the articles on diseases of the liver, pancreas, kidneys, etc. The former articles in the present edition have been considerably extended, and new articles on direct laryngoscopy, bronchoscopy and œsophagoscopy, by Mr. Waggett, and on diseases of the trachea, by Sir Felix Semon and Dr. Watson Williams, have been inserted. Also the subject of diseases of the ear has been dealt with in a series of articles by several writers under the general supervision of Dr. M'Bride. As now arranged, the book before us is a complete and authoritative treatise upon diseases of the throat, nose and ear, written by well-known specialists, and as such will appeal to many readers outside the circle of those more particularly interested in purely medical subjects, and we commend the wisdom of the editors in deciding to devote one complete part of the *System* to these subjects, a part which is separately obtainable. The utility of the work is enhanced by many coloured plates, which are in all respects excellent.——Vol. VI. Pp. xvi, 861. London: Macmillan and Co. Limited. 1909.—In this edition the editors have wisely devoted one whole volume, and one volume only, to the discussion of circulatory disorders. This is a great improvement, without any drawbacks, upon the arrangement which in the first edition gave parts of two volumes to these subjects. Some changes in the authorship of the articles have inevitably occurred. Dr. Dreschfeld and Dr. A. E. Sansom have died, and their contributions on acute simple endocarditis and diseases of the mitral valve have been revised by Dr. Thomas McCrae, of Baltimore, and Dr. George A.

Gibson. Professor W. H. Welch's articles on thrombosis and embolism have been brought up to date by Dr. Rolleston, and the introductory section on the physics of the circulation, originally written by Professor Sherrington, has been entrusted to Dr. James Mackenzie, whose influence on the study of cardiac disease finds further expression in a number of graphic records scattered throughout the book. There is one quite new section on Stokes-Adams Disease, by Professor Osler and Dr. Arthur Keith, which constitutes the best account of the subject in medical literature. Sir Clifford Allbutt has rewritten his article on "Overstress of the Heart," including much valuable evidence as to the effect of athletic effort upon the heart, collected by Dr. R. W. Michell, of Cambridge. The learned writer's opinion is to the effect that muscular exertion has been over-estimated as a factor in cardiac strain. Most of the sections are admirably up to date, and it is difficult to find any omission of importance, except in one or two which are perhaps unduly conservative. The whole volume is a practical contradiction of the theory that medical writers must take no account of style; it is delightful writing throughout, from the direct, business-like paragraphs of Professor Osler to the leisurely, classical essays of his Cambridge colleague. It is, indeed, an admirable example of the width of outlook and soundness of judgment for which English medicine is justly noted.

**The Causation of Sex.** By E. Rumley Dawson. Pp. xii, 196. London: H. K. Lewis. 1909.—This little work, quite apart from the author's theory, is well worthy of perusal on account of the very exhaustive account of the work of different authors on reproduction, and the number of interesting and curious cases quoted. As regards the theory itself, in spite of the evidence produced by the author in its favour, we must still maintain a non-committal attitude, and on the whole regard it as non-proven. The interesting cases quoted by the author would be convincing were it not that the numbers quoted are so small in relation to the number of male and female births respectively that the possibility of the occurrences being merely coincidences cannot be lost sight of. We do not consider that the case of the woman who bears a family of both male and female children after unilateral ovariotomy or öophorectomy can be dismissed so easily as the author seems to assume by saying that in every such case portions of ovarian tissue were left behind; so many of these cases are on record that we should be justified by the author's argument in saying that ovarian tissue always exists in the utero-ovarian ligament, or that all operators are more or less inexpert. If this is the case, how is it that bilateral öophorectomy produces menstrual cessation, which is shown by the author to be practically identical with cessation of ovulation? Obviously it cannot be assumed that all bilateral oophorectomies are perfect operations if all unilateral are imperfect ones. The margin of difference which may enter into the statistical presentation of a calculation in which the total numbers are approximately equal is obviously very wide, as witness the falling of heads or tails in a large number of tosses. In a few hundred such examples, without the law of averages being in the least disturbed, a run of one variety may occur, which may seem to prove that one or other is more frequent; and although the author's evidence is interesting, and his deductions well drawn, we consider that to prove that one ovary produces males and one females requires much larger statistical figures.

**Consumption, its prevention and home treatment: a guide for the use of patients.** By H. Hyslop Thomson, M.D. Pp. 75. London: Oxford Medical Publications. 1910.—The educational influence of the sanatorium is greatly aided by the literature proceeding therefrom. Dr. Thomson, writing from the Liverpool Sanatorium at Frodsham, remarks that "not only does it aid the consumptive in his personal conflict with the disease, but it takes an active part in the great war of prevention, which has its battle-ground in the homes of the people." The principles and practices of open-air home treatment are described, chiefly for the benefit of those who are waiting for admission, and also for those who, having left the sanatorium, wish to continue home treatment on the lines of a well-conducted sanatorium for consumptives. This little book of 75 pages gives an excellent outline of what the consumptive requires.

**The Expectation of Life of the Consumptive after Sanatorium Treatment.** By Noel D. Bardswell, M.D. Pp. 130. London: Oxford Medical Publications. 1910.—The medical superintendent of the King's Sanatorium at Midhurst here gives his quota of evidence as to the value of sanatoria for consumptives. The evidence consists of the careers of 241 patients discharged during the years 1900 to 1905, whose subsequent life history is recorded. They indicate that permanent recovery may be looked for in from 70 to 80 per cent. of cases of early consumption, in from 40 to 60 per cent. of cases of moderately advanced disease, and in some 8 to 10 per cent. only of the much advanced cases. The author concludes that "sanatorium treatment must be regarded as a relative cure only. With great reservations . . . it can be considered to be curative in a high degree. As regards the far advanced cases, it must be recognised as a palliative, and as a means of prolonging life." It postpones the inevitable indefinitely so far as tuberculosis is concerned.

**The Influence of Strong, Prevalent, Rain-bearing Winds on the Prevalence of Phthisis.** By William Gordon, M.A., M.D. Pp. xiv, 108. London: H. K. Lewis. 1910.—This subject has engaged the author's attention for a period of ten years, and he gives in this volume the collected evidence in favour of his view that strong, rain-bearing winds have a prejudicial effect on phthisis. Commencing his observations by a careful analysis of the prevalence of phthisis in the rural districts of Devonshire, he found that the mortality was nearly five times as great in the parishes exposed to west and south-west winds as amongst the sheltered parishes. Again, the streets of Exeter gave a similar result, inasmuch as the death-rate in those streets which were exposed to south-west, west, or north-west winds was found to be twice, or even four times as great as that in the streets sheltered from these winds. By extending his investigations farther afield, he found that from all quarters of the globe he obtained extraordinary confirmation of the conclusions derived from the home area. A careful consideration of the facts obtained leads to the conclusion that such a mass of evidence, carefully sifted, yet so constant in its indications, cannot be set aside as fallacious.

**Mind and Health: the Mental Factor and Suggestion in Treatment, with special reference to Neurasthenia and other common nervous disorders.** By Edwin Ash, M.D. Pp. 116. London: H. J. Glaisher. 1910.—Another handbook for students and practitioners of medicine who require a brief account of the practical uses of suggestion in treatment, and for students of psychology who are interested in the application of that subject to the healing art. Books of this kind, and better ones, are so numerous that we cannot understand for what purpose this one was written.

**Nature's Help to Happiness, or Ground Treatment.** By John Warren Achorn, M.D. **Nervousness: a brief and popular review of the moral treatment of disordered nerves.** By A. T. Schofield, M.D. London: Rider's Mind and Body Handbooks. 1910.—It would be a misfortune that any neurasthenic patient should read these books, as they would effectually prevent any endeavours of the physician to cure by suggestion. We think that a thrilling novel would be far more useful. For whom, then, are they intended?

**Air and Health.** By Ronald C. Macfie, M.A., M.B. Pp. vii, 345. The New Library of Medicine. London: Methuen and Co.—The book deals with the physical and chemical properties of air, particularly with reference to health and diseases. The physiology of respiration is considered in its practical bearings, and chapters are devoted to the question of climate and to relevant questions of dust, fog, germs, air-borne epidemics, etc. Ventilation is fully discussed, both in its private and public aspects. Dr. Macfie has written several most interesting books. This, his latest, is rather from the point of view of a sanatorium physician, and accordingly he concludes with a chapter on the open-air treatment of consumption and open-air schools. He believes that "nothing will so increase the happiness, the health, the moral and mental sanity of the nation as open-air schools." Two very complete indexes of names and of subjects complete a volume which is full of interesting details and replete with knowledge of all kinds. There is not a dull page from cover to cover.

**Aseptic Surgery.** By CHARLES BARRETT LOCKWOOD, F.R.C.S. Third Edition. Pp. xii, 275. London: Oxford Medical Publications. 1909.—This book is too well known for much to be said as to its contents. The details of surgical technique are expounded by one who has been in the forefront of asepsis since its application to modern surgery. The first edition was the first English discourse on the subject which really attracted the attention of the mass of operators to the principles of Listerism. It was so complete that it has been necessary to add but little since. We are rather surprised that the preparation of the skin by iodine, which is very extensively used under certain circumstances, has not been mentioned, and the same remark applies to the preparation of catgut by the iodine method or by boiling in Xylol. All questions of asepsis are a consideration of details, and anyone who wishes to look these up cannot do better than consult this work.

**A Synopsis of Surgery.** By E. W. HEY GROVES, M.S., M.D., B.Sc., F.R.C.S. Second Edition. Pp. viii, 579. Bristol: John Wright and Sons Ltd.—No book on surgery could be more condensed than this one is and at the same time contain such an excellently arranged array of facts. The author has thoroughly covered the whole ground. It is well balanced throughout, no section being given undue prominence. A student just going up for an examination has often difficulty in finding out his weak points; with this in his hand he can rapidly run through the whole range of surgery, and on finding some subject on which he feels doubtful he can easily refer to a larger work. Treatment, and especially after-treatment, is dealt with very tersely; the latter is a subject examiners are constantly asking. There is no mention of when patients may get up after operations, or when a fractured leg may be put to the ground. No method of treating fractured patella is mentioned except wiring "For deformities of the legs after rickets has ceased osteotomy will generally be required" seems to us a little drastic, and the same might be said of a radical mastoid operation for acute mastoiditis. The book is everywhere up to date, and should be very useful for revision work.

**Emergencies of General Practice.** By PERCY SARGENT, M.B., B.C., and ALFRED E. RUSSELL, M.D., B.S. Pp. xi, 364. London: Oxford Medical Publications. 1910.—This book claims to be a collation of facts for easy reference and a record of personal experience. Its form is good, the illustrations are of variable excellence, and it includes subjects which can hardly be regarded as emergencies of general practice, such as the operative treatment of gastric hemorrhage, fractured patella, and general peritonitis. It will form a useful addition to the numerous works of reference of its class.

**The Nutrition of the Infant.** By Ralph Vincent, M.D. Third Edition. Pp. xxii, 342. London: Baillière, Tindall and Cox. 1910.—That this book has in seven years reached its third edition is a welcome proof of the interest taken in the feeding of infants by the medical profession in this country. Dr. Vincent writes with authority about matters which are constantly being put into practice in the treatment of the patients under his care at the Infants' Hospital. The book is a detailed exposition of the Rotch system of feeding infants by milk, the exact proportions of whose principal ingredients are stated in prescriptions which vary according to the individual needs of each baby; a scientific system as opposed to rule-of-thumb methods. Dr. Vincent has carried out his task with the greatest thoroughness; and his book, though a shade too scientific and too insistent on the particular method he advocates, is the best exposition of that method and its applications which we possess.

**Handbook of Diseases of the Ear.** By Richard Lake, F.R.C.S. Eng. Third Edition. Pp. xii, 248. London: Baillière, Tindall and Cox. 1910.—The third edition of this excellent handbook has been largely rewritten as well as increased in size, so as to be virtually a new book, and as the additions include most of the modern advances in aural surgery, it gives us in a handy form a valuable guide to the present-day knowledge of diagnosing and treating diseases of the ear. Among the additions we notice a useful table of six subdivisions of Rinne's test, by which we may group the various conditions affecting the hearing of a deaf person, and make them pathognomonic of those affections; a good account is also given of oto-massage and its various methods of application, especially in sclerosis, for which the author specially recommends it. The book contains some excellent coloured plates as well as numerous original illustrations, some of which we notice were from drawings supplied by Mr. Harold F. Mole.

**Errors of Refraction.** By Chas. Blair, M.D., F.R.C.S. Second Edition. Pp. 106. Bristol: John Wright & Sons Ltd. 1910.—A small straightforward exposition of the subject, but too academic to be of much use to the practitioner who knows but little of the methods and who essays to test for glasses. The author would have greatly added to its value if he had included a chapter on the common pitfalls which a beginner is so liable to come across, such as overlooking very faint, old nebulæ, and spending useless time in trying to do a retinoscopy, or ordering presbyopic glasses to a patient without recognising early cataract, and many others which no doubt would be readily called to mind.

**Landmarks and Surface Markings of the Human Body.** By Louis Bathe Rawling, M.B., B.C., F.R.C.S. Third Edition. Pp. viii, 96. London: H. K. Lewis. 1908.—The third edition improves on the second by the insertion of several new illustrations. The author has resisted the temptation to enlarge the scope of the work, and we think is to be commended for doing so, as this is one of the best small readable text-books on the subject, and will prove very useful and reliable.

**Cerebral Congestion and Tight Neck Clothing.** By Walter G. Walford, M.D. Pp. 26. London: H. K. Lewis. 1910.—This booklet, written by a retired medical man of large experience, calls attention to an insidious cause of many disorders. That neck constriction may be a cause of many troubles other than those of the head is the contention of the author, and he gives many cases in proof of his theorem. The loosening of a button is so easy, and the discomfort of tightness is so decided, that one needs little persuasion to adopt his theory and the practice resulting therefrom.

**Henry Phipps Institute.** Philadelphia. 1908 and 1909.—The fourth and fifth annual reports of this Institute for the study, treatment and prevention of tuberculosis contain accounts of the general and special clinical and pathological work done by members of the staff at the Institute during the years. They are edited by Dr. Joseph Walsh, and are a record of much good work in a variety of subjects, *e.g.* the bone marrow in pulmonary tuberculosis, the opsonic index, fibrosis, pneumothorax, etc.

**"The Prescriber."** Vol. iii. Edinburgh: 137 George Street. 1909.—This monthly journal, dealing with therapeutics, pharmacology and the newer remedies, edited by Thos. Stephenson, Ph.C., F.C.S., is the latest addition to our exchange list, and we welcome it as an aid towards the almost impossible task of eliminating the good from the useless of the new remedies which are constantly pouring in from the makers and venders of new products. We are told that the ingenuity of the makers of new remedies during 1908 seems largely directed towards the invention of new names; this is harmless in itself, but if the new name is simply a disguise for a familiar drug in order that it may be obtained from one source only, then these names may well be forgotten and ignored. Secret remedies are not to be encouraged, and this journal will do all in its power to favour legislation restricting the traffic in such goods. We wish the editor all success in his endeavours, and our readers will find much useful information in the pages of this new exchange.

